# Effectiveness of personalized face-to-face and telephone nursing counseling interventions for cardiovascular risk factors: a controlled clinical trial^1^

**DOI:** 10.1590/1518-8345.0626.2747

**Published:** 2016-08-08

**Authors:** Vivian Vílchez Barboza, Tatiana Paravic Klijn, Alide Salazar Molina, Katia Lorena Sáez Carrillo

**Affiliations:** 2 PhD, Professor, Escuela de Enfermería, Universidad de Costa Rica, Montes de Oca, San José.; 3 PhD, Full Professor, Facultad de Enfermería, Universidad de Concepción, Concepción, Chile.; 4 PhD, Associate Professor, Facultad de Enfermería, Universidad de Concepción, Concepción, Chile.; 5 PhD, Associate Professor, Facultad de Ciencias Físicas y Matemáticas, Departamento de Estadística, Universidad de Concepción, Chile.

**Keywords:** Clinical Trial, Risk Factors, Cardiovascular Nursing, Quality of Life, Directive Counselling

## Abstract

**Objective:**

to evaluate the effect and gender differences of an innovative intervention involving in-person and telephone nursing counseling to control cardiovascular risk factors (arterial hypertension, dyslipidemia, and overweight), improve health-related quality of life and strengthen self-efficacy and social support in persons using the municipal health centers' cardiovascular health program.

**Method:**

a randomized controlled clinical trial involving participants randomized into the intervention group who received traditional consultation plus personalized and telephone nursing counseling for 7 months (n = 53) and the control group (n = 56). The study followed the Consolidated Standards of Reporting Trials Statement.

**Results:**

women in the intervention group presented a significant increase in the physical and mental health components compared to the control group, with decreases in weight, abdominal circumference, total cholesterol, low-density lipoprotein cholesterol, and the atherogenic index. The effects attributable to the intervention in the men in the intervention group were increased physical and emotional roles and decreased systolic and diastolic pressure, waist circumference, total cholesterol, low-density lipoprotein cholesterol, atherogenic index, cardiovascular risk factor, and 10-year coronary risk.

**Conclusion:**

this intervention is an effective strategy for the control of three cardiovascular risk factors and the improvement of health-related quality of life.

## Introduction

The World Health Organization considers noncommunicable diseases (NCDs) to be the leading causes of death and disability worldwide, with cardiovascular diseases being one of the principal NCDs(1). In Chile, cardiovascular diseases (ischemic heart disease and cerebrovascular disease) are the leading causes of mortality(2). Additionally, they are also one of the leading causes of disability and impaired quality of life. Hypertension and dyslipidemia are common risk factors in addition to the corresponding lifestyle factors. In Chile, the Cardiovascular Health Program (Programa de Salud Cardiovascular, PSCV) has contributed to an increase in the coverage of people with arterial hypertension and to greater control of arterial pressure (AP) and total cholesterol levels(3). However, cardiovascular diseases are still prevalent and cardiovascular risk factors continue to increase(4), probably due to the way in which the provision of care is presented. 

Studies are in agreement that cardiovascular diseases are strongly related to lifestyle and biological risk factors(5). Therefore, intervention studies measure cardiovascular risk factors such as cholesterol, systolic pressure, body mass index (BMI), diet, and physical activity levels in addition to health-related quality of life (HRQoL), self-efficacy, and social support(6). Moreover, health care for these diseases should recognize the biological peculiarities of each gender, including differences in cardiovascular risk factors in terms of both prevalence and the way they are presented via different pathophysiological mechanisms in men and women(7), because these factors influence the specific HRQoL diagnosis of these patients.

Evidence points to the effectiveness of nursing interventions that combine in-person methodology with telephone interventions(8). Moreover, studies have suggested that it is the responsibility of nurses to implement strategies that contribute to the control of modifiable risk factors for cardiovascular disease(9) and have reported significant improvements in AP, cholesterol, BMI, physical activity, and feeding indicators in the intervention groups(10).

In Chile, a recent intervention performed by nurses implemented and evaluated a telephone support model for the self-management of chronic disease (apoyo telefónico para el auto-manejo de enfermedad crónica -ATAS) that was initiated in public primary care centers for people with diabetes mellitus type 2(11). The results showed that the intervention improved the care of the PSCV controls, stabilized the glycated hemoglobin levels, and decreased the ingestion of unhealthy foods. The perception of self-efficacy also increased.

## Objective

To evaluate the effect and gender differences of an innovative intervention involving in-person and telephone nursing counseling to control cardiovascular risk factors (arterial hypertension, dyslipidemia, and overweight), improve the HRQoL, and strengthen self-efficacy and social support in persons using the cardiovascular health program of the municipal health centers in Concepción.

## Methods 

Design and type of study: This study was a randomized controlled clinical trial that followed the Consolidated Standards of Reporting Trials Statement. Population: There are 8 health centers in Concepción. Six of these centers are dependent on the municipality and serve 72% of the beneficiary population of the National Health Fund (Fondo Nacional de Salud- FONASA); the other 2 centers are dependent on the Health Service and serve 28% of the population. The 6 centers sharing the same dependency were considered for this study. A total of 640 men and women between 35 and 64 years of age who were registered and validated at the municipal Family Health Centers (Centro de Salud Familiar -CESFAM) and the PSCV in Concepción with three sets of risk factors (hypertension, overweight, and dyslipidemia) formed the population of this study.

The population was selected from the electronic records of the clinical files of each of the 6 CESFAMs. Subsequently, the individuals were contacted to verify their willingness to participate in the research. A total of 224 individuals met the inclusion criteria because no sampling was performed. Of these, 120 agreed to participate in the study and signed the informed consent form.

Inclusion criteria: Individuals between 35 and 64 years of age, bearing three cardiovascular risk factors, and residing in the sector in which they were enrolled. The exclusion criteria were defined based on the fact that this study was an intervention aimed at primary prevention to prevent people from developing diabetes and/or metabolic syndrome. The criteria were as follows: people who did not have the three risk factors described or people with ischemic cardiopathy, cerebrovascular accident with sequelae, chronic obstructive pulmonary disease, any mental illness or dementia, alcoholism, terminal illnesses, immobilization, thyroid disease, cancer, human immunodeficiency virus/AIDS, or severe rheumatic disease.

**Figure 1:**
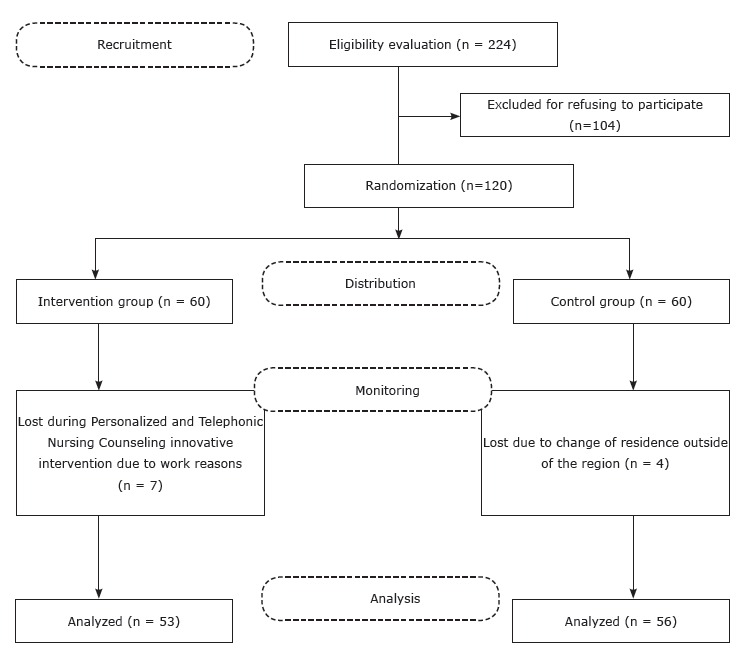
Flowchart of the research

Next, the pre-test measurement was performed. Randomization: To form groups from the 120 participants, a hierarchical cluster analysis using the Mahalanobis distance and Ward's algorithm was performed by gender (females, n = 82 and males, n = 38) based on the initial homologation variables (AP, BMI, gender, HRQoL, general self-efficacy, and perception of social support). A total of 60 individuals were randomly selected for the control group and 60 for the intervention group. When applying the respective tests, no significant differences were found between the control and intervention groups. At the end of the study, the intervention group was composed of 53 people (13 men and 40 women) who completed all nursing counseling sessions during the 7-month period and the post-test measurement. In the control group, 56 people (20 men and 36 women) completed the post-test measurement. Both groups continued to receive traditional care provided by the Health Center PSCV. Figure 1 presents the study flowchart.

Instruments: The biosociodemographic data questionnaire compiled by the researchers (the generic Health-Related Quality of Life Questionnaire SF-36(12)) contained 36 questions that were grouped into 8 dimensions of health, which in turn were aggregated into two summary measures as follows: the physical health component (PHC) and the mental health component (MHC). The scores range from 0 to 100, with 0 being the worst result and 100 the best. The syntactic and semantic adaptations of the instrument for the country were performed in Chile in 2006 and applied to a representative sample of the adult population (> 15 years of age) that were the beneficiaries of the public and private health systems of Chile with a Cronbach's alpha of 0.7(13). The General Self-Efficacy Scale(14) is composed of 10 items. The answers are Likert-type with a minimum score of 10 points and a maximum of 40 points as follows: incorrect (1 point), barely true (2 points), mostly true (3 points), or true (4 points). A higher score indicates greater general self-efficacy. The use of this scale in Chile was published in 2010(15); this study indicated that the scale was reliable and valid for the measurement of the perceived self-efficacy construct in the Chilean population with a Cronbach's alpha of 0.84. The Multidimensional Scale of Perceived Social Support (MSPSS)(16) is composed of 12 Likert-type response items with 4 alternatives for each item (1 = almost never, 2 = sometimes, 3 = often, and 4 = always or almost always). A higher score indicates the presence of greater social support. This scale was validated for the 12 items in the region of La Araucanía by Ortiz & Baeza(17). The Cronbach's alpha reliability coefficient was 0.87.

BMI(18) was measured with a scale and stadiometer. Arterial pressure(19) was measured with a mercury sphygmomanometer and stethoscope. The abdominal circumference was measured using a tape measure with calipers(18). The lipid profile was measured using blood chemistry tests in a clinical laboratory, including total cholesterol (Col-Total), high-density lipoprotein (Col-HDL), low-density lipoprotein (Col-LDL), triglycerides, atherogenic index (LDL/HDL), and cardiovascular risk factor (TC/HDL). The 10-year coronary risk was calculated based on the Framingham Tables, which were adapted to the characteristics of the Chilean population following a standard procedure(20). The data corresponding to the application of the instruments were collected by a volunteer from the health field and a senior year nursing student after training by the researchers. The anthropometric measurements, AP, and 10-year cardiovascular risk factor calculation were conducted by the nursing student. Statistical analysis: The data analysis was performed using SAS OnlineDoc(r), version 9.2 (SAS Institute Inc., Cary, NC, USA, 2003), with descriptive and inferential statistics. The data were subjected to the distribution analysis using the Shapiro-Wilk test. The variables that presented normal distributions were analyzed using Student's t-test, whereas those that did not present normal distributions were analyzed using the Mann-Whitney U test. For the comparison of paired groups, the Wilcoxon test and the paired t-test for groups were used according to the type of distribution. To distinguish the association between categorical variables, the Chi-squared test or Fisher's exact test were used (when the expected frequency values less than 5 accounted for more than 25% of the cells). The level of significance used was α = 0.05.

## Intervention 

Nursing counseling is understood to be an existential process that focuses on the meaning of the person's health-related life experiences and then becomes a process of dialogue(21) based on effective communication and face-to-face support. From this perspective, feelings, thoughts, and attitudes are explored and expressed to clarify behavior or conduct in relation to a particular health situation and thus aid in decision-making(21). The counseling has HRQoL as its central axis and is designed based on the health needs of the target population with the incorporation of self-efficacy and social support. For its development, an integrative bibliographic review of the period between 1994 and 2013 was performed. The databases reviewed were Web of Science, ScienceDirect, PubMed, MEDLINE, SciELO, LILACS, and the printed journals in the Library of the School of Medicine of the University of Concepción and the Library of Nursing at the Pontifical Catholic University of Chile. The reviewed studies agree that cardiovascular diseases are strongly related to physical inactivity, overweight/obesity, smoking, high cholesterol levels, hypertension, and diabetes(5), making it clear that the risk factors are characteristics or behaviors of individuals who increase the likelihood of cardiovascular disease(3). The results underscore the hypothesis that interventions, programs, and health services managed and performed by nurses are effective in primary health care because they offer an appropriate response to the needs of the population; thus, it is necessary to perform more research aimed at impacting the health-disease process in the individual user and the influence on their quality of life(22). Furthermore, the principles of the theory of Human Becoming were contemplated for the development of this intervention (23).

**Figure 2:**
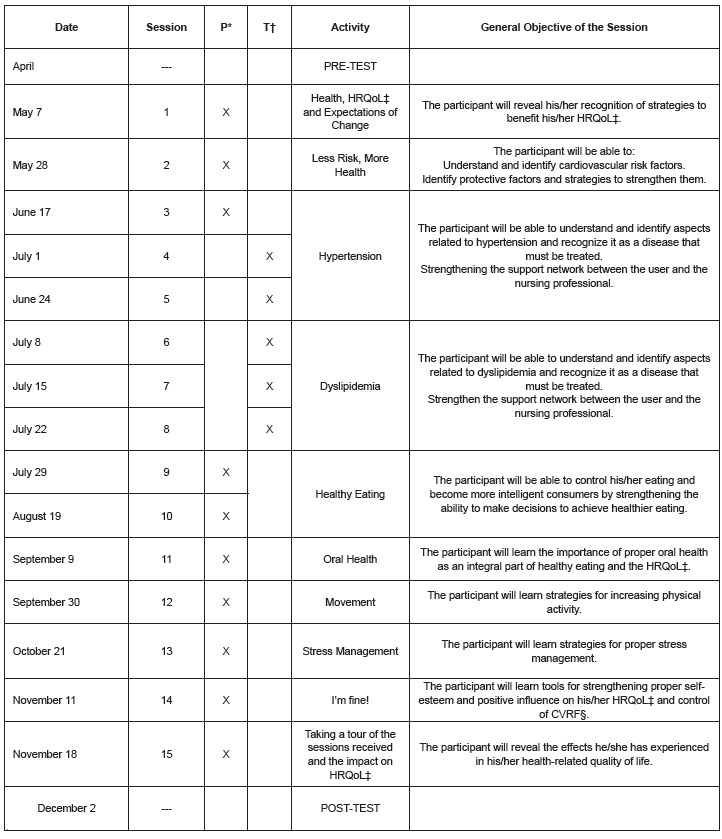
Sessions performed in the Innovative Intervention

A total of 15 sessions were proposed, including 10 in-person sessions with a maximum duration of one hour and 5 telephone sessions of 15 minutes each. The sessions were developed by the researchers(24), who had access to the data after the study was completed. The telephone sessions were assigned as reinforcements of the issues on which the users received some education in the PSCV. The issues worked on in the counseling sessions were the same for men and women, although the order in which they were addressed differed depending on the expectations of change of each participant. For example, the women placed a priority on emotional issues and the men on physical issues. Another difference was the average duration in minutes of the sessions. The sessions were approximately 40 minutes for men and 60 minutes for women. The distribution of the sessions is shown in Figure 2:

*P: Personalized, †T: Telephonic, ‡HRQoL: Health-Related Quality of Life, §CVRF: Cardiovascular Risk Factor.

The selection of similar variables in both groups allowed comparisons between the groups, which demonstrated that the intervention results were reliable. To this end, the population was randomized and homologated. None of the participants changed medical treatment or CESFAM during the months of the study, thereby decreasing possible factors that could cause confusion or modification of the effect. The same monitoring and measurement methods were used for all study participants to achieve comparability of the information. The study was blinded to the participants and there was no significant differential loss of participants.

## Ethical aspects 

This research was approved by the Bioethics Committee of the School of Medicine of the University of Concepción, Chile (registration DIFM 042/2012), and was authorized by the Directorate of Health Administration of Concepción. The ethical requirements of E. Emmanuel were considered, including the signing of an informed consent form. The study was performed in the Obesity and Overweight Prevention Unit (Unidad de Prevención de la Obesidad y el Sobrepeso- UPOS), which forms part of the University of Concepción. 

## Results 

Table 1 gathers the characteristics of the 109 people with three cardiovascular risk factors who attended a PSCV in one of the six municipal CESFAMs of Concepción and who answered the data collection instrument in the pre-test for the control group and intervention group. The table shows that both groups are comprised of a higher percentage of women and that most participants are in the age range between 60 and 64 years with a high school education and are married; the nuclear family predominated.

**Table 1:** Biosociodemographic characteristics of the participants. Concepción, Chile 2013

Variable			Group			Total			Stadigraph	gl	p-value
			Control		Intervention		n=109				
			n=56		n=53						
			F	%	F	%	F	%			
Gender											
	Female		36	64.3	40	75.5	76	69.7	1.61*	1	0.2039
	Male		20	35.7	13	24.5	33	30.3			
Age in completed years											
	Age range	35-44 years	1	1.8	2	3.8	3	2.8	2.160†	2	0.354
		45-54 years	23	41.1	14	26.6	37	33.9			
		55-64 years	32	57.2	37	69.8	69	63.3			
Last level of schooling attended/graduated											
	Basic Education		10	17.9	10	18.8	20	18.3	7.62*	8	0.4713
	High School Education		29	51.8	28	52.8	57	52.3			
	Technical Education		8	14.3	11	20.8	19	17.4			
	University Education		9	16	4	7.6	13	11.9			
Current marital status											
	Single		20		22		42		0.386*	1	0.534
	Cohabitating		36		31		67				
Type of family											
	Alone		3	5.4	6	11.3	9	8.3	1.29†	2	0.583
	Nuclear		34	60.7	31	58.5	65	59.6			
	Extended family		19	33.9	16	30.2	35	32.1			
Current occupational situation											
	Employed		31		27		58		0.213*	1	0.644
	Inactive Unemployed		25		26		51				

* Chi-squared test, † Fisher's exact test

As shown in Tables 2 and ^3^, both strategies (the traditional consultation by the PSCV and the nursing counseling provided in the study) showed progress in most variables. However, the intervention group achieved significant changes. Table 2 shows that the women in the intervention group presented significant improvements compared to the control group in weight, abdominal circumference, Col-Total, Col-LDL, LDL/HDL, the two summary measures of the HRQoL (the physical and mental health components), body pain, social function, and vitality dimensions between the pre-and post-test measurements. Table 2 shows that the women in the experimental group presented a greater increase in the scores for general self-efficacy and perceived social support compared to the control group. However, this change was not significant.

**Table 2:** Effect of the innovative intervention: personalized and telephonic nursing counseling for cardiovascular risk factors in women in the control group and intervention group. Concepción, Chile 2013

Women		Control Group			Intervention Group			Effect of the Intervention (95% Confidence Interval)	Stadigraph	p-value
		Pre-test	Post-test	Pre- and Post-test difference	Pre-test	Post-test	Pre- and Post-test difference			
Variables										
	Physical Health Indicators									
	Col-Total	178.2	176	2.22	201.1	178.7	22.4	-20.18 (-36.42; -3.94)	2.48*	0.0156†
	Col-HDL	51.8	52.3	-0.5	52.5	50.6	1.93	-2.43 (-4.52; -0.33)	2.31*	0.0237‡
	Col-LDL	105.3	104.1	1.19	123.4	103.1	20.24	-19.05 (-33.48; -4.61)	2.63*	0.0104†
	LDL/HDL	2	2	0.04	126	2.4	2.1	-0.25 (-0.5; 0.00)	1.97*	0.0527‡
	Triglycerides	105.7	98.1	7.67	126	124.7	1.23	6.44 (-17.47; 30.35)	1384§	0.9834
	TC/HDL	3.5	3.4	0.09	3.9	0.7	0.35	-0.26 (-0.56; 0.03)	1260§	0.1899
	10-year Coronary Risk	2.4	0.8	0.81	2.7	0.8	0.88	-0.07 (-0.49; 0.35)	1361.5§	0.7848
	Systolic Pressure	129.2	133.4	-4.28	133.2	131.6	1.63	-5.9 (-12.16; 0.35)	-1.88	0.0641
	Diastolic Pressure	89.6	77.1	12.47	91.5	78.9	12.63	-0.15 (-5.14; 4.84)	-0.06	0.9515
	Weight	65.4	65.3	0.08	67.3	66.4	0.94	-0.85 (-1.7; -0.01)	1192§	0.0435‡
	Abdominal Circumference	93.1	92.3	0.84	95.8	91.5	4.35	-3.51 (-5.27; -1.75)	1001.5§	0.0001†
	Body Mass Index	27.6	27.6	0.05	28.1	27.7	0.38	-0.33 (-0.69; 0.02)	1204§	0.0583
Health-Related Quality of Life										
Physical Health Component		77.1	81	-3.93	79.7	88.9	-9.2	5.27 (0.16; 10.38)	2.06*	0.0433‡
	Body Pain	56.9	60.3	-3.34	60.8	77	-16.25	12.92 (2.52; 23.31)	2.48*	0.0155†
	Physical Function	86.3	90.5	-4.16	89.6	94.3	-4.67	0.5 (-3.01; 4.01)	1439.5§	0.5739
	Physical Role	93.1	94.8	-1.73	94.4	98.4	-4.06	2.33 (-5.11; 9.77)	1466.5§	0.3152
	General Health	72	78.5	-6.48	73.9	85.8	-11.83	5.35 (-2.91; 13.61)	1520§	0.1624
Mental Health Component		68.5	73.3	-4.82	66.9	78.9	-11.95	7.14 (1.21; 13.06)	2.40*	0.0189†
	Social Function	82.5	86.7	-4.17	80.3	95	-14.75	10.58 (0.96; 20.2)	1582§	0.0349‡
	Emotional Role	54.3	57.2	-2.96	52	58.7	-6.67	3.7 (-1.54; 8.95)	1495.5§	0.2074
	Mental Health	70.8	78.3	-7.55	70.5	84.9	-14.4	6.84 (-2.27; 15.96)	1.50*	0.1386
	Vitality	66.4	71	-4.58	65	77	-12	7.42 (0.17; 14.67)	2.04*	0.0451‡
General Self-efficacy		34.3	36.8	-2.5	36	38.4	-2.37	-0.13 (-2.3; 2.05)	0.11*	0.9091
Perceived Social Support		36.5	38.1	-1.58	35.9	39.7	-3.8	2.22 (-6.5; 1.9)	1472.5§	0.3660

* t-test for paired groups, † p ≤0.01, ‡ p ≤0.05, § Wilcoxon test

**Table 3:** Effect of the innovative intervention: personalized and telephone using counseling for cardiovascular risk factors in men in the control group and intervention group. Concepción, Chile 2013

Men		Control Group			Intervention Group			Effect of the Intervention (95% Confidence Interval)	Stadigraph	p-value
		Pre-test	Post-test	Pre- and Post-test difference	Pre-test	Post-test	Pre- and Post-test difference			
Variables										
Physical Health Indicators										
	Col-Total	175.7	177.1	-1.4	190.5	157.3	33.23	-34.63 (-59.89; -9.38)	2.80*	0.0088†
	Col-HDL	43.3	43.3	0	43.6	41.2	2.38	-2.38 (-5.48; 0.71)	1.57*	0.1258
	Col-LDL	107.7	109.7	-2.03	113.5	87.3	26.25	-28.28 (-48.45; -8.11)	2.86*	0.0075†
	LDL/HDL	2.5	2.6	-0.13	2.6	2.1	0.49	-0.63 (-1.05; -0.21)	298§	0.0045†
	Triglycerides	123.6	120.9	2.65	167	144	23	-20.35 (-59.02; 18.32)	1.07*	0.2919
	TC/HDL	4.1	4	0.05	4.4	3.8	0.54	-0.49 (-0.94; -0.04)	2.23*	0.0033‡
	10-year Coronary Risk	3.5	3.2	0.3	4.7	3.1	1.62	-1.32 (-2.6; -0.03)	271.5§	0.0546‡
	Systolic Pressure	135.1	139.7	-4.55	141.9	132.2	9.77	-14.32 (-24.78; -3.85)	-2.79	0.0089†
	Diastolic Pressure	91.8	83.6	8.20*	99	81.1	17.92	-9.72 (-18.00; -1.44)	-2.4	0.0228‡
	Weight	78.3	78.2	0.05	81.5	80.4	1.06	-1.02 (-2.68; 0.65)	244§	0.3966
	Abdominal Circumference	96.6	96.8	-0.23	101.5	97	4.50	-4.73 (-7.03; -2.42)	4.17*	0.0002†
	Body Mass Index	27.5	27.5	0.03	28.4	28.1	0.37	-0.34 (-0.92; 0.25)	243§	0.4176
Health-Related Quality of Life										
Physical Health Component		80.5	86.5	-6.03	84.4	90.3	-5.85	-0.18 (-5.63; 5.27)	0.07*	0.9462
	Body Pain	60	78.5	-18.5	73.1	77.7	-4.62	-13.88 (-31.96; 4.19)	268§	0.0735
	Physical Function	92.7	96	-3.33	89.2	95.9	-6.67	3.33 (-3.35; 10.02)	183§	0.1531
	Physical Role	94.4	92.5	2.88	97.1	100	-2.88	5.77 (-2.88; 14.42)	189§	0.0546‡
	General Health	75	79.2	-4.17	78.2	87.4	-9.23	5.06 (-4.39; 14.51)	1.09*	0.2832
Mental Health Component		72	77.3	-5.26	78.7	81.3	-2.57	-2.69 (-8.63; 3.26)	0.92*	0.3638
	Social Function	85.5	91	-5.5	99.2	95.4	3.85	-9.35 (-18.53; -0.16)	264.5§	0.0791
	Emotional Role	49.7	58	-8.33	59	58	1.03	-9.36 (-16.68; -2.03)	277.5§	0.0131†
	Mental Health	78.2	83.4	-5.2	82.5	91.1	-8.62	3.42 (-6.29; 13.12)	0.72*	0.4784
	Vitality	74.8	76.8	-2	74.2	80.8	-6.54	4.54 (-4.86; 13.94)	0.98*	0.3324
General Self-efficacy		36	37.7	-1.65	37.5	39	-1.46	-0.19 (-3.18; 2.8)	238§	0.5227
Perceived Social Support		37	38.7	-1.7	38.7	41.7	-3	1.3 (-4.67; 7.27)	0.44*	0.6602

* t-test for paired groups, † p ≤0.01, ‡ p ≤0.05, § Wilcoxon test

Table 3 shows that the men in the intervention group presented significant changes compared to the control group in abdominal circumference, Col-Total, Col-LDL, TC/HDL, LDL/HDL, 10-year coronary risk, systolic and diastolic pressure, the physical role dimension of the physical health component and the emotional role dimension of the mental health component between the pre- and post-test measurements. In the intervention group, there was a highly significant improvement in abdominal circumference compared to the control group between the pre- and post-test measurements. With respect to general self-efficacy and perceived social support in men, we observed a greater increase in the scores of both variables in the experimental group compared to the control group. However, this change did not achieve significance as was observed for the women.

## Discussion 

The data from this study show a predominance of females. This finding is consistent with the results obtained in a Spanish study(25), which showed that differences between the genders in cardiovascular risk factors, such as arterial hypertension and dyslipidemia, were higher in women, influenced by social class, and accentuated by age. By categorizing the variable of age in completed years into ranges, a higher percentage was observed in the groups of people 55-64 and 45-54 years of age. The largest number of people with hypertension are within these ranges(25). The prevalence of cardiovascular disease was reported to increase with age; additionally, the risk profile in women was reported to be greater than the risk profile in men(26). The counseling intervention had a significant effect on increasing the HRQoL in the women in the intervention group. This result can be attributed to the empowerment and strengthening of the support networks and the actions of the nurse him/herself during the development of the counseling. In contrast, no significant differences in the HRQoL were observed in the men participating in the study. This result is consistent with the result reported by a study on the effectiveness of an intervention program for weight control in people with hypertension that observed that although the HRQoL improved in the intervention group members, the differences were not significant(27). The nursing intervention had no significant effect on general self-efficacy and perceived social support in either women or men despite an increase in the scores of the intervention group compared to the control group. This result could be explained by the characteristics of the studied pathologies or the measurement instruments. The measurement of the general self-efficacy scale results in a contradiction between the high scores of the baseline measurement and the participants' experiences during the development of the counseling because the changes in the experimental process are not reflected in the post-test measurement. In the women, weight and abdominal circumference decreased significantly, whereas only the abdominal circumference decreased in the men. The effect obtained in the anthropometric measurements may have been motivated by the use of a pedometer in counseling. The use of a pedometer in primary care interventions has been reported to lead to an increase in the physical activity of the users together with the social support provided by the health professional(28). The results obtained concerning the AP levels of the women participating in this study showed the need to prolong the nursing intervention because significant changes were achieved in these variables after 18 months(29). Unlike the women, the men exhibited significantly decreased systolic pressure and diastolic pressure. This result is consistent with an intervention performed for 6 months aimed at people with excess weight and hypertension where the reduction of AP was significant in men(30). This finding can be analyzed from the social function because men receive family support, especially from their partner, who begins to modify their style of cooking and eating patterns. This process is different for women because they have to cook differently for themselves and the rest of their family because the family members do not want to follow her diet. A significant decrease in the lipid profile was attributable to the intervention in both the female and male participants. This finding could be due to the incorporation of healthy eating and increased physical activity into the lifestyles of the participants. Furthermore, the present investigation showed an effect on the reduction of the 10-year coronary risk only in the males. No significant changes were detected in the triglyceride levels in either group after 12 months of intervention(29). The results obtained in this study showed that implementing personalized and telephone nursing counseling for cardiovascular health can be the basis for preventive interventions for cardiovascular disease and the promotion of health. Despite the positive effects produced by this nursing intervention, we recommend allowing more than seven months to develop this modality of intervention and to establish this type of differentiated nursing care by gender as a continuous process over time because one year is a short time in which to achieve long-term changes and evaluate the results(31). However, significant changes were achieved in both men and women with the continued development of this intervention that augur greater effects. The intervention was performed in a personalized manner, in-person and by telephone, to respond to the call made to nursing as a discipline for innovative efforts to contribute to the challenge of halting cardiovascular disease through interventions that develop interactive, culturally relevant models and incorporate the specific contexts of each individual in order to achieve behavioral changes in people with chronic diseases(32). 

The limitations in the study are that the instruments used did not quantify the social support provided by the nurse or the self-efficacy of the management of the chronic disease. Atherosclerosis is recognized as a risk factor and other emerging risk factors exist. However, these variables were not measured in this study due to time and economic resource constraints. Despite these limitations, the results were found to be valid by comparing changes between the women and men in the control group with the intervention group. These results indicate that this type of nursing counseling favors the control and reduction of cardiovascular risk factors and improves the health-related quality of life in people with hypertension, overweight, and dyslipidemia.

## Conclusion 

The innovative intervention of in-person and telephonic nursing counseling aimed at controlling cardiovascular risk factors (arterial hypertension, dyslipidemia, and overweight), improving the HRQoL, and strengthening self-efficacy and social support resulted in a significant decrease in abdominal circumference (AC), Col-Total, Col-LDL, and TC/HDL in women and men. Only women exhibited significantly decreased weight and an increased HRQoL. The men exhibited significantly decreased systolic blood pressure (SBP) and diastolic blood pressure (DBP), LDL/HDL, and 10-year coronary risk. The women and men in the control group presented no significant changes. 

The counseling service confirmed the need to dedicate between 30 and 40 minutes for each man and between 45 and 60 minutes for each woman for the nursing consultation to clarify all doubts and check the understanding of patients concerning issues related to their health status. Furthermore, we demonstrated the importance of establishing an interactive consultation that establishes a bond of trust and active listening with the user to enable feelings of support from the nursing professional during the health care process. Finally, we concluded that this type of in-person and telephonic personalized nursing counseling intervention was effective in controlling the cardiovascular risk and improving the health-related quality of life in men and women with hypertension, overweight, and dyslipidemia.
